# The Role of Translational Regulation in Survival after Radiation Damage; an Opportunity for Proteomics Analysis

**DOI:** 10.3390/proteomes2020272

**Published:** 2014-06-11

**Authors:** Stefanie Stickel, Nathan Gomes, Tin Tin Su

**Affiliations:** 1 Department of Molecular, Cellular and Developmental Biology, University of Colorado, Boulder, CO 80309, USA; E-Mails: stefanie.stickel@gmail.com (S.S.); nathan.gomes@colorado.edu (N.G.); 2 SuviCa, Inc. P O Box 3131, Boulder, CO 80301, USA

**Keywords:** ionizing radiation, ribosome, translation

## Abstract

In this review, we will summarize the data from different model systems that illustrate the need for proteome-wide analyses of the biological consequences of ionizing radiation (IR). IR remains one of three main therapy choices for oncology, the others being surgery and chemotherapy. Understanding how cells and tissues respond to IR is essential for improving therapeutic regimes against cancer. Numerous studies demonstrating the changes in the transcriptome following exposure to IR, in diverse systems, can be found in the scientific literature. However, the limitation of our knowledge is illustrated by the fact that the number of transcripts that change after IR exposure is approximately an order of magnitude lower than the number of transcripts that re-localize to or from ribosomes under similar conditions. Furthermore, changes in the post-translational modifications of proteins (phosphorylation, acetylation as well as degradation) are profoundly important for the cellular response to IR. These considerations make proteomics a highly suitable tool for mechanistic studies of the effect of IR. Strikingly such studies remain outnumbered by those utilizing proteomics for diagnostic purposes such as the identification of biomarkers for the outcome of radiation therapy. Here we will discuss the role of the ribosome and translational regulation in the survival and preservation of cells and tissues after exposure to ionizing radiation. In doing so we hope to provide a strong incentive for the study of proteome-wide changes following IR exposure.

## 1. The Effect of Ionizing Radiation on Cells and Tissues

### 1.1. DNA Damage Responses: Cell Cycle Checkpoints, DNA Repair and Cell Death

**Figure 1:**
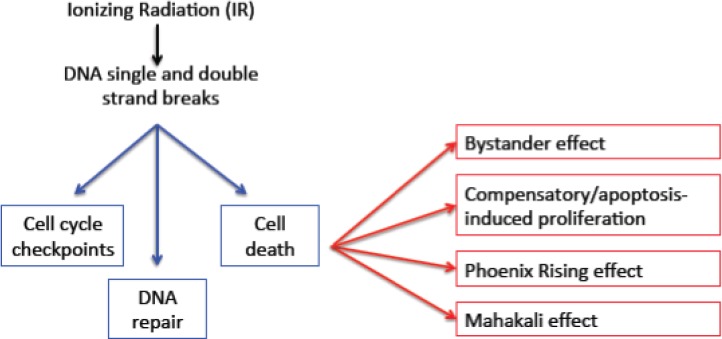
DNA damage responses that would be amenable to proteomic analysis. Ionization Radiation (IR) causes DNA single and double strand breaks that result in three canonical responses: activation of cell cycle checkpoints, induction of DNA repair and cell death by apoptosis (blue arrows and boxes). In metazoa, dying cells exert non-autonomous effects (red) that include the radiation bystander effect, compensatory or apoptosis-induced cell proliferation, the Phoenix Rising effect and the Mahakali effect. All of these responses involve alterations in the proteomes through changes in transcription, translational regulation, post-translational modification and protein degradation. See text for details.

Ionizing radiation (IR) collectively refers to radiation (X-rays and γ-rays) and high-energy particles (alpha and beta particles) with sufficient energy to break not only chemical bonds but also atoms into ions [1]. The most prominent consequences to IR-treated cells are DNA single and double-strand breaks (DSB) [2,3,4]. The preponderance of these breaks occur following DNA interaction with reactive oxygen species generated by the ionization of water, and to a lesser extent by the direct cleavage of phosphodiester bonds by IR [1]. If left unrepaired DSBs are highly lethal; therefore, cells employ a plethora of responses collectively known as DNA damage responses (DDR) to efficiently repair these DNA lesions. Canonical DDR encompasses the combination of several key cellular responses including cell cycle arrest by checkpoint activation, DNA repair and/or cell death (typically via apoptosis). DDR has been described in several excellent reviews (e.g., [2,5,6,7]), and will not be covered in detail here. However we will briefly discuss the secondary consequences of IR-induced cell death in the following paragraphs as this appears to be important for the survival of tissues and organisms (Figure 1).

### 1.2. Secondary Consequences of Cell Death

In metazoans the behavior of neighboring cells influences the others. The consequences of such interactions vary from developmental cell fate determination to life and death choices following genotoxic insult. For example, in the larvae of *Drosophila melanogaster*, dying cells emit mitogenic signals to stimulate their neighboring cells to proliferate. Depending on the tissue context, these signals act through Wingless (Drosophila Wnt), JNK, Hh (Hedgehog), or tumor suppressor Salvador/Warts/ Hippo pathways [8,9,10,11]. Increased proliferation of the neighbors is thought to help compensate for the dying cells and to contribute to tissue regeneration. A similar but mechanistically distinct phenomenon was also identified in mammalian systems. The “Phoenix Rising” pathway occurs in murine tissue following IR induced cell death. Here, activated caspases 3 and 7, required for apoptosis, also mediate the activation of phospholipase A_2_ and the subsequent production and release of prostaglandin E_2_. These signals act non-autonomously to stimulate proliferation and tissue regeneration [12]. In a follow-up study, caspase 3 was found to be necessary for tumor regeneration after radiation treatment in mouse xenograft models [13]. 

In the context of disease, tumors have been demonstrated to undergo “accelerated repopulation” following radiation therapy, resulting in increased tumor growth rate [14]. Accelerated repopulation, in which cells that survived irradiation proliferate even faster than cells prior to irradiation, was first documented in transplantable murine fibrosarcomas; radiation treatment increased tumor growth relative to no treatment [15]. Additionally single dose titrations of IR in transplantable rhabdomyosarcomas demonstrated that the rate of tumor repopulation is correlated with the IR dose. Finally, evidence of accelerated repopulation has also been demonstrated in head and neck, cervical, and non-small cell lung cancers [1,14,16,17], as well as being implicated in the failure of chemo-radiation therapy combinations in patients [18]. As such, the typical radiotherapy regimen consists of multiple fractionated doses, each scheduled to curb accelerated repopulation after the preceding dose [18]. While the exact mechanisms of accelerated repopulation are not well understood, IR treatment has been demonstrated to induce cellular proliferation through epidermal growth factor receptor (EGFR) activation [18]. Interestingly, the mitogenic effect of radiation damage may be widely conserved through evolution; the pathogenic fungus *Wangiella dermatitidis* also shows increased growth (increased cell size and increased division) following exposure to low doses of IR [19]. 

In addition to the mitogenic effects described in the studies above we have documented a non-autonomous protective (anti-apoptotic) phenomenon in irradiated dying cells in Drosophila larvae [20]. This “Mahakali effect” is dose-dependent as increasing amounts of cell death leads to larger protected regions. The Mahakali effect requires the receptor tyrosine kinase Tie (homolog of Tie-1 and Tie-2 in mammals) and can be blocked by the expression of the caspase inhibitor p35 in dying cells [20,21]. The requirement for caspase activity makes the Mahakali effect similar to the mammalian Phoenix Rising effect, because, in both cases, caspase activity in dying cells is required for the release of mitogenic signals [12]. 

The effects neighboring cells have on one another are not always protective or mitogenic. For example, in the “radiation bystander effect” described in mammalian cell culture and mice, irradiated cells make their neighbors more prone to death [22,23,24]. Antioxidants such as L-deprenyl and lactate can inhibit the bystander effect [25], suggesting that oxidative stress and energy metabolism may be involved.

The studies described to this point indicate that IR exposure can set into motion multiple primary and secondary cellular responses, some of which function by cell non-autonomous mechanisms. Many of these responses involve post-translational modifications of proteins, protein degradation (e.g., caspase cleavage during apoptosis and degradation of caspase targets during Phoenix Rising and Mahakali effect) and altered protein synthesis as described in the next section. Examination of these changes is highly suitable for proteomic analysis. In fact, one could argue that proteomic analyses are required to fully understand short and long-term consequences of IR exposure. Yet, systemic studies of radiation responses typically address changes in the transcriptome rather than the proteome. It is our hope that this review will provide strong motivation for increased proteomic analyses of IR responses.

### 1.3. The Effect on Macromolecules

We note that ionizing radiation can damage not only DNA but also other macromolecules in the cell such as proteins, RNAs and lipids. In fact, damage to membrane lipids occurs after IR exposure and may have a role in signaling through the generation of ceramide [26,27]. A discussion of lipidomes, however, is outside the scope of this review on proteomes. Similarly, protein carbonylation the oxidation of amino acid side chains is a well-known outcome of IR. In bacteria the level of protein carbonylation correlates with radiation sensitivity [28]. In eukaryotes, the level of carbonylation varies and a functional role remains to be determined [29]. 

## 2. Translational Regulation in Response to IR

Regulation of protein synthesis is critically important for the generation of proteins required for cell growth, proliferation and survival [30,31]. This is particularly true following exposure to IR as cells must generate proteins required for DNA repair, survival and recovery. In this section, we summarize eukaryotic translational mechanisms and how they are regulated in response to IR exposure. 

### 2.1. Mechanisms of Translation

**Figure 2:**
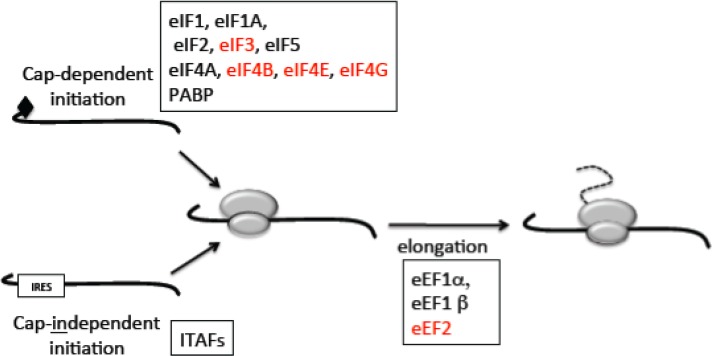
Eukaryotic translation initiation and elongation. Protein factors required are shown in the box next to each step. Of these, the ones known to be regulated or modified following exposure to ionizing radiation are in red font. The diamond represents the tri-methyl-G cap on the mRNA. Internal ribosome entry site (IRES) represents the internal ribosome entry site used in cap-independent initiation. The mRNA (black line) is depicted as linear for the sake of clarity, but would in reality be circularized as the 5' and the 3' ends interact via proteins bound. The ovals represent the ribosome and the dashed line represents the nascent polypeptide chain. ITAFs = IRES transacting factors. PABP = Poly A binding protein. The termination step is not shown. See text for details.

Translation occurs in three distinct stages: initiation, elongation, and termination (Figure 2). Under normal conditions, initiation is rate-limiting and involves the assembly of the pre-initiation complex (PIC) and the mRNP complex [32]. The PIC is composed of a sub-complex of Met-tRNA_i_-eIF2-GDP, initiation factors eIF1, eIF1A, eIF 3, and eIF 5, and the small 40S ribosomal subunit. Generally, *de novo* protein synthesis is “cap-dependent”, requiring a 5' mRNA tri-methyl-guanine cap used to assemble the mRNP complex that is composed of circularized mRNA, initiation factors eIF4A, eIF4B, eIF4E, and eIF4G, in addition to the poly A-binding protein (PABP) [33,34,35,36,37]. The PIC and the mRNP associate to form the 43S-mRNA complex, which scans for the start (AUG) codon. Once the start codon is reached, the 60S ribosomal subunit joins to form the 80S ribosome complex and initiate elongation.

In contrast to cap-dependent initiation, cap-independent initiation requires only a subset of eIFs and utilizes a structured internal ribosome entry site (IRES) located in the 5' UTR of mRNAs which is capable of binding some of the factors that participate in canonical translation initiation [38]. Because ribosomes do not scan IRES-containing mRNAs, IRES trans-acting factors (ITAFs) help position the ribosome on the start codon. Furthermore, IRES-mediated initiation does not utilize the eIF4E initiation factor which helps mediate the rate-limiting step of cap-dependent initiation.

In contrast to initiation, a single known mechanism mediates translation elongation. Elongation begins when GTP-bound elongation factor 1 α (EF1α) brings an amino acyl tRNA to the ribosome [39]. EF1α then undergoes GTP-hydrolysis and leaves the ribosome in a GDP state. The guanine nucleotide exchange factor (GEF) EF1β (not shown in Figure 2) helps catalyze the exchange of GDP for GTP on EF1α so it may be used again. Next, the peptidyl transferase of the 60S ribosomal subunit catalyzes the transfer of the growing peptide chain from the peptidyl tRNA to the amino acid of the incoming amino acyl tRNA. This results in the partial translocation of both tRNAs within the ribosome. Elongation factor 2 (EF2) hydrolyzes a GTP to fully translocate the tRNAs within the ribosome, facilitating the departure of the used tRNA from the ribosome and making room for the next amino acyl tRNA to bind the ribosome. There is currently no known GEF of EF2. This process will continue cycling until a stop codon is reached and termination will occur with the help of release factors.

### 2.2. The Switch to Cap-Independent Initiation under Stress

Limited cap-independent initiation occurs in homeostatic cells, for example, during mitosis and differentiation; however it becomes the predominant form of translation during various forms of cellular stress (e.g., IR, hypoxia, lack of nutrients) [38]. As protein synthesis is the most energy intensive cellular process [40], translation regulation is tightly coupled to cellular growth and stress [34,37,40,41]. Consequently inhibition of global protein synthesis can save energy in times of cellular stress. As such inhibition of the essential initiation factor eIF4E, at least in part, blocks cap-dependent initiation during stress. Conversely, approximately 10% of all mRNAs contain an IRES [37,41]. Many of these mRNAs encode proteins required for cell growth, cell proliferation and survival. Therefore, while global translation can be blocked by inhibition of cap-dependent initiation, translation of cap-independent mRNAs involved in survival can still occur, making the latter the predominant mode of translation under stress.

### 2.3. Mechanisms for Regulation of Translation

The phosphatidylinositol 3-kinase (PI3K)/Akt/mammalian target of rapamycin (mTOR) signaling pathway acts to integrate extracellular signals and nutritional status with cellular growth. Among the effectors of PI3K/AKt/mTOR signaling are most of the components of translation initiation, including the rate-limiting factor eIF4E [42]. In response to growth stimuli, PI3K is activated, which subsequently activates Akt. In turn Akt inhibits the tuberous sclerosis complex 1/2 (TSC1/2), an inhibitor of Rheb, a Ras-like GTPase. Uninhibited Rheb then activates mTOR, which then regulates three key components involved in translation initiation: eIF4E, eIF4G, and S6 kinase (S6K) [37,42,43,44,45]. mTOR phosphorylates and represses the eIF4E-binding protein 1 (4E-BP1) and induces its dissociation from eIF4E. Free eIF4E can associate with eIF4G for the initiation of cap-dependent translation. Additionally, mTOR stimulates the interaction between eIF4G (part of the mRNP complex) and eIF3 (part of the PIC) to enhance translation [46]. Lastly, mTOR can phosphorylate S6K, which in turn phosphorylates eIF4B and the ribosomal subunit S6 to stimulate translation. Therefore, mTOR signaling can directly and indirectly modulate cap-dependent translation initiation. 

Modulation of protein synthesis in response to stress may not only be only limited to translation initiation. Recent studies indicate that the EF2 kinase (EF2K), which phosphorylates and inactivates EF2, is modulated in response to genotoxic stress [47]. For example, as osteosarcoma cells (U2OS) recover from doxorubicin treatment EF2 is de-repressed by degradation of EF2K. Interruption of this regulatory pathway disrupts cellular recovery suggesting that regulators of translation elongation are required for successful recovery from genotoxic stress. In addition, EF2K is also subject to mTOR regulation [48,49], indicating that mTOR has the potential to regulate translation elongation in addition to initiation.

### 2.4. The Evidence for Translational Regulation after IR: Changes in Transcriptome vs. Ribosome-Associated Transcriptome

Evidence for translational regulation in response to IR comes from several ribosome-profiling studies. In these cases, overall changes in mRNA associated with polysomes, representing actively translated mRNAs, is profoundly greater than compared to genome-wide changes in total transcripts. For example, in glioblastoma cells numerous mRNAs are recruited to and from polysomes 6 hours after IR exposure [50]. Side-by-side comparison of changes to the transcriptome and changes in the polysome profile in this study demonstrated that 10 times more mRNAs showed altered translation than transcription. That is approximately 100 mRNAs showing at least a 2-fold change in levels (increase or decrease), whereas more than 1000 mRNAs showed at least a 2-fold change in polysome association (increase or decrease). Categories of mRNAs whose polysome profiles changed included those involved in cell proliferation, transcription, as well as nucleotide and RNA metabolism. Additionally, analysis of protein levels showed that most but not all mRNAs (14 of 16 analyzed) whose polysome profile changed showed a corresponding change in protein levels. This suggests that in addition to altered synthesis, post-translational controls such as protein stability contribute to changes after irradiation.

A similar study that examined glioma, breast, lung and pancreatic cancer cells also showed profound changes in polysome profiles after irradiation [51]. Interestingly, mRNAs that showed significant changes in polysome profiles clustered based on the tissue of origin. For example, “cell-to-cell signaling”, “cell assembly”, and “cellular movement” were among the top functional clusters affected in breast cancer cells. Conversely “cell death” was among the top functional clusters for lung cancer and glioma. Corresponding datasets on protein levels in such experiments would be invaluable and would help us fully understand the DNA damage responses.

### 2.5. Potential Mechanisms for Altered Polysome Profiles: The Effect of IR on Translation Factors

Following exposure to IR, multiple mechanisms appear capable of inhibiting translation factors that participate in cap-dependent initiation (red fonts in Figure 2). As described earlier, mTOR plays a vital role in regulating protein synthesis. Additional studies have established that this role can be extended to controlling translation in response to IR. For example, IR exposure inhibits cap-dependent translation through c-Abl, a protein that can bind and inhibit mTOR [52]. Further examination revealed that this was due to enhanced binding of 4E-BP1, an mTOR effector, to eIF4E, an essential initiation factor. As c-Abl is activated in response to DNA damage induced by IR treatment, it is hypothesized that this may be a mechanism by which genotoxic stress triggers inhibition of cap-dependent translation and induction of cell cycle arrest. Another mechanism for the inhibition of eIF4E following IR exposure has also been documented; via p53- and ATM-mediated increase in 4E-BP1 protein levels [34]. 

In murine fibroblasts and human leukemia cells, IR exposure activates p21-activated kinase 2 (Pak2, gamma-Pak) [53]. Further, expression of wild-type Pak2 in rabbit reticulocyte lysates and cultured cells has been shown to inhibit translation, while kinase-inactive mutants have no effect. Because Pak2 has the ability to phosphorylate, bind, and inactivate eIF4G, it is hypothesized that translation inhibition is due to the decreased association between eIF4G and eIF4E, which is required for cap-dependent translation [54]. This matches with other studies demonstrating reduced association of eIF4G and eIF4E following IR exposure [55]. Finally, caspase-dependent degradation of eIF4G1, eIF3A, and eIF4B has been reported in irradiated Jurkat E6 T-lymphoma cells [37]. Taken together these observations provide for mechanisms in which inhibition of cap-dependent initiation can occur following IR exposure, while cap-independent initiation remains largely unaffected.

HuR is an RNA-binding protein that has been shown to bind and regulate the stability and translation rate of many mRNAs encoding proteins for cell proliferation, cell death and stress responses. IR exposure alters the mRNAs that associate with HuR and these changes are dependent on the ATM kinase, a key mediator of DDR [56]. As a result this mechanism has the potential to alter the profile of mRNAs in polysomes following IR.

MDM2 (mouse double minute 2), a protein classically known for mediating p53 degradation appears to have a direct role in translation [57]. MDM2 was found to bind the IRES of XIAP mRNA and promote the translation of this anti-apoptotic protein. After IR exposure, MDM2 localizes to the cytoplasm and promotes the translation of XIAP1. The resulting increase in XIAP1 protein levels increases resistance of human leukemia cells to IR-induced apoptosis.

Finally, translation termination factor eRF3 was recently shown to be a target of caspase-mediated degradation following exposure to UV and etoposide, a topoisomerase II inhibitor, in multiple cell types [58]. This raises the possibility that eRF3 degradation following IR exposure may block translation termination, thereby altering the profile of mRNAs in polysomes. Taken together these data demonstrate that multiple levels of post-transcription protein expression are regulated in response to IR induced stress.

### 2.6. Interfering with Translation Compromises Radiation Responses

It has been known for some time that inhibition of protein synthesis modifies radiation responses. For example, in HeLa cervical cancer cells, protein synthesis inhibitors decreased the survival of cells exposed to X-rays [59]. In another example, “pre-irradiation” of human lymphocytes with low doses (1cGy) of X-rays made cells less sensitive to subsequent X-ray exposure [60]. However, if cells are treated with cycloheximide, a protein synthesis inhibitor, 4 to 6 hours after the initial dose, the protective response was inhibited. These studies suggest that translation of pro-survival factors, such as repair enzymes, occurs after irradiation and can offer protection against subsequent radiation exposure. 

These observations stand in contrast with other findings that inhibition of protein synthesis following irradiation inhibited cell death. For example, pre-treatment of MOLT-4 human lymphoblast-like cells with cycloheximide inhibits γ-radiation induced apoptosis [61]. The protective effect of translation inhibitors against X-ray-induced apoptosis was also seen *in vivo* in rats [62,63]. Collectively, in these studies, translation was required for IR-induced cell death. It remains possible that differences in cell type (prone to repair *vs.* prone to death, for example) and experimental protocols (as, for instance, inhibition of protein synthesis before or after IR exposure) made a difference in whether translation inhibition was pro-survival or pro-death in these studies. Nonetheless, we can conclude that new protein synthesis was necessary for the proper response to IR in these experimental systems. Given the role of PI3K signaling in regulating protein synthesis, interfering with this pathway might be expected to compromise radiation responses. In fact, a recent study shows that inactivation of PI3K/TOR signaling correlated with premature senescence following low dose IR exposure in cultured human endothelial cells [64].

## 3. Prior Studies Using Proteomic Analysis after IR Exposure

We summarized in preceding sections how the profile of mRNAs in polysomes is altered following exposure to ionizing radiation. It follows that the amount of protein being synthesized will also be profoundly altered. However, the situation may be more complicated than that. This is due in part to the fact that the levels of cellular proteins reflect not only rates of synthesis but also rates of degradation. There are few studies in which polysome profiles and proteome composition are compared in parallel. In one study the effects of the proteasome inhibitor bortezomib upon the transcriptome (by RNAseq), translation (by polysome profiling) and the proteome (by mass spectrometry) were measured. Despite significant changes in the transcriptome and in the polysome profile, the resulting changes to the proteasome were modest [65]. In another study, polysome profiling and ribosome foot-printing were used to identify a large number (>300 total) of mRNAs that became differentially translated during egg activation in Drosophila [66]. However, proteomic analysis of the corresponding samples by quantitative mass spectrometry after stable isotope labeling of peptides (peptide dimethylation) showed that changes in protein level correlated poorly with either changes in polysome recruitment (R = 0.17) or changes in translation efficiency (R = 0.2). Such findings illustrate the need to analyze the proteome in order to fully understand the consequences of an experimental manipulation. Indeed radiation proteomics is an active field. Recent reviews collectively provide a comprehensive account of prior studies [67,68].

Prior studies suggest that low doses of IR (0.2–1 Gy) may yield proteomic responses that are sensitive to experimental design and chance variation and are often contradictory [68]. The following sections focus on studies that use higher doses (two or more Gy per fraction on cultured cells, for example) that are considered more clinically relevant. 

### 3.1. Proteomic Studies of DDR Responses

ATM kinases are key mediators of DNA damage responses in diverse eukaryotes that range from yeast to humans. Human patients with mutations in ATM show hypersensitivity to radiation and are cancer prone. The availability of patient-derived cell lines that have been complemented with ATM cDNA provides isogenic cells with and without ATM for functional analyses. Proteomic analysis of these cells following exposure to X-rays illustrated both ATM-dependent and ATM-ndependent changes [69]. These differences were identified using 2D gel electrophoresis followed by spot identification through matrix assisted laser desorption/ionization-time of flight, time of flight (MALDI-TOF-TOF) spectrometry. Additionally virally immortalized patient derived fibroblasts expressing ATM cDNAs were treated with irradiation. This resulted in an increase of proteins involved in stress pathways, cell cycle arrest, protein degradation and apoptosis. Conversely, the levels decreased for proteins involved in metabolic pathways, cell proliferation and cell differentiation. In the isogenic ATM deficient counterparts these changes were attenuated, illustrating their dependence on ATM activity. Interestingly, many of the changes still occurred but with delayed and dampened levels, indicating secondary mediators of the DNA damage response. 

In addition to detecting changes in bulk protein levels, it is of particular interest to use mass spectrometry to detect post-translational modifications (e.g., phosphorylation, acetylation, etc) of the proteome, as they have the ability to modulate protein function without affecting protein abundance. For example, in virally-transformed B-lymphocytes, 594 out of 5204 sites with a detectable phosphorylation status showed 2-fold or greater change following irradiation [70]. Phospho-peptides were enriched by electrostatic repulsion-hydrophilic interaction chromatography (ERLIC) and TiO(2)-based protocols and analyzed by mass spectrometry. SILAC (stable isotope labeling by/with amino acids in cell culture) was used to distinguish control and experimental samples. Sequence analysis identified SQ (target of ATM/ATR kinases) and SXXQ (a new sequence determinant) as the most common consensus phosphorylation sites. Interestingly, changes included not only transient or lasting increases in phosphorylation (some up to 8 hours) but also transient or lasting de-phosphorylation events. In fact, dephosphorylated sites constituted as much as a third of all sites with changes, suggesting a significant role of de-phosphorylation in DDR.

Many proteins with key roles in DDR are not only regulated by phosphorylation but also by acetylation, with p53 being a well-known example [71]. In a human osteosarcoma cell line (U2OS), 40 out of 204 sites with a detectable acetylation status showed a greater than 1.5-fold change in acetylation following exposure to 6 Gy of X-rays. Again SILAC was used to distinguish control and experimental samples, in this case by enriching using an anti-acetylated-lysine antibody. The majority of observed changes involved transient deacetylation, occurring within 5 min of irradiation, and full recovery occurring within 60 min [72]. The transient nature of changes in phosphorylation and acetylation poses a challenge that could be met by following these changes over a time course.

Unlike the case of phosphorylation, no consensus amino acid sequence was observed for sites of acetylation. The majority of affected proteins had roles in chromatin and chromosome organization based on gene ontology. Indeed, changes in the acetylation of histones constituted the majority of observed modifications. Interestingly, acetylation changes occurred on the eukaryotic translation elongation factor 1 alpha-like protein 7, whose role is not understood but is suspected to be involved in translation. As a result, secondary consequences to the proteome via altered translation are possible. While both of these studies by Bennetzen and colleagues focused on nuclear proteins, we propose that expansion of such analyses to cytoplasmic proteins would be highly informative.

An important issue to consider in these studies concerns the choice of “fold cut-off”, that is, whether a 1.5-fold or a 2-fold change should be considered significant. While a consensus value may be appealing in principle, it may not be the most biologically meaningful. This is because a 2-fold change in protein X may not affect its function while a 1.5-fold or lower change in protein Y could have profound biological consequences [67]. Instead, we espouse the view that a greater need exists for (1) making complete sets of raw data easily accessible in addition to highlighting hits that meet whichever criteria the authors choose and (2) assessing the biological significance of candidates in independent loss or gain of function studies.

### 3.2. Searches for Biomarkers for Radiotherapy Pharmacodynamics and Side Effects

Our survey of the literature revealed that the most frequent use of proteomic analysis after irradiation is to identify correlations with the effect of radiation in clinical settings or in quality control (e.g., [73]). For example, gamma irradiation with high doses (25–50 Gy) before storage is the standard protocol to eliminate T-lymphocytes from red blood cell concentrates. The removal of T-lymphocytes helps prevent graft-versus-host reactions during transfusion. To study the long term effect of high dose IR, red blood cells (RBCs) were irradiated with 30 Gy, and proteins were differentially dye-labeled and then separated by 2D gel electrophoresis [74]. Protein spots that showed differences between irradiated and controls were identified by in-gel digestion followed by mass spectrometry (LC-MS/MS). Only modest changes in the RBC proteome at 1 and at 15 days after irradiation were found as 27 of 2392 spots showed changes but none by more than 2-fold. These results support the hypothesis that standard practices do not alter the quality of RBCs, but what is more interesting is the suggestion that significant effects of IR are only possible in the presence of an active genome. RBCs, of course, are enucleate and lack the ability to transcribe or translate in response to IR.

In another study, proteomic analysis was performed to identify proteins that correlated with a major side effect of radiation therapy, fatigue. Sera from patients receiving external beam radiation therapy for non-metastatic prostate cancer were subject to 2D gel electrophoresis followed by LC-MS/MS analysis. Identification of altered spots showed that apolipoprotein A and E as positively correlating with and transthyretin negatively correlating with fatigue [75]. In an analogous study, male infertility was addressed using whole body irradiation in mice as a model system [76]. The method of 2D gel electrophoresis followed by MALDI-TOF-TOF mass spectrometry analysis of sperm from mice 7 days post-carbon ion beam irradiation identified 5 proteins whose level correlated with sperm motility. As sperm motility affects fertility, these proteins may be potential biomarkers for fertility after radiation therapy in human.

Thoracic radiation exposure during the treatment of breast and lung cancers leaves the heart as a critical off-target. In an attempt to identify cardiac damage following radiation mice were treated with 8 and 16 Gy single doses of X-rays, in order to mimic the total radiation dosage normally given at approximately 2 Gy per fraction, applied multiple times per week, for two to three weeks [77]. Cardiac tissues were isolated after 16 weeks and subjected to differential isotope coded protein labeling (ICPL) of tissue lysates thereby allowing control and experiment peaks to be distinguished by mass spectrometry. LC-ESI-MS/MS Analysis identified 33 proteins whose expression changed with both doses of radiation. Of these, approximately half was up-regulated, while the rest down-regulated. Proteins with altered levels included those involved with energy and lipid metabolism, antioxidant defense and cytoskeletal organization. Many of these were mitochondrial proteins. Further analysis identified the transcription factor complex, peroxisome proliferator-activated receptor (PPAR), as a potential mediator of these changes and, consequently, identified a potential drug target for curbing radiation damage in the heart.

Radiation is the standard of care for non-operable non-small-cell lung cancer. Traditional therapy consists of low radiation doses given multiple times (e.g., 2 Gy daily for several weeks). However, dosing schedules are changing with the advent of stereotactic delivery methods that can “split” the radiation beam into multiple fractions that intersect at the tumor. Utilizing this method, larger ablative doses (e.g., 3 fractions of 18 Gy) are delivered to the tumor while the surrounding tissues are exposed to only a fraction of this dose. However the collateral effects of these new treatment regiments remains unclear. Therefore, a recent study focused on proteomic changes occurring in cancer-associated fibroblasts (CAFs) after radiation exposure [78]. CAFs are suspected of secreting paracrine factors following radiation exposure that promote tumor cell survival. Supernatants of cultured CAFs following ablative radiation doses were subject to one-dimensional electrophoresis followed by tandem mass spectrometric analysis. The results suggest that ablative radiation doses alter the secretory profile of CAFs with respect to some, but not all, growth factors. Although these factors were found to have only mild effects upon the growth and migratory behaviors of co-cultured cells, this study does provide a framework for delineating the non-autonomous cell growth effects that may arise following irradiation of tumors.

The un-biased and comprehensive nature of proteomic analysis makes it highly suitable for identifying biomarkers such as those correlating with success or failure of radiotherapy. Further application of this technique is likely to produce additional markers in, for example, different cancer types. Indeed, many candidate markers have been identified in studies of head and neck, breast, prostate and rectal cancers [73]. We see two challenges going forward. First, there is likely to be significant individual-to-individual variation; therefore, validating each marker in multiple patients will of paramount importance. Second, these analyses will identify correlations, but much more work will be needed to understand the causal relationship between a given marker and radiation exposure. Such an understanding will be necessary in order to improve the effectiveness of radiotherapy.

## 4. Opportunities: Unanswered Questions that Will Benefit from Proteomics Analysis

Multiple lines of evidence suggest that protein synthesis is not only mis-regulated but also contributes to diseases for which IR is a therapeutic module. Overexpression of eIF3, eIF4G, or eIF4E leads cells to develop oncogenic properties and/or undergo cellular transformation [79,80,81,82,83,84]. Furthermore, overexpression of eIF4E increases the translation of weakly translated mRNAs that contain a structured, GC rich 5' UTR, which encode proteins involved in growth, angiogenesis, and tumor invasion [85,86,87,88]. Conversely, down-regulation of initiation and elongation factors eIF3, eIF4G, eI4E, and EF1α, led to decreased oncogenic properties in various cancer cell lines [83,89,90,91]. Furthermore, suppression of eIF4E in a mouse xenograft model showed tumor reduction without toxicity [86]. These data suggest that altered translation is central to cancer initiation and progression. Given that IR is a main therapy module for cancer, understanding how IR alters translation and the resulting proteome is of utmost importance. Yet, application of proteomic analysis to radiation biology of cancers remains in the early stages as described in this review.

We envision several specific areas that could benefit from proteomic analysis. A time course of proteomic changes through initial DDR responses and later consequences of cell death in multi-cellular settings, where cell-non-autonomous effects are prevalent, would be particularly informative. These could be applied to established cell lines or patient derived samples, *in vitro* or in animals, as well as in model organisms.

A major application of proteomics could be to answer the question of how caspase activity results in cell death after irradiation. Presumably, caspases act by cleaving their targets. A comprehensive proteomic analysis after experimental activation of each caspase could be informative. Along these lines, the role of protein degradation in DDR remains to be fully understood. Caspases are the obvious proteases active in cells destined to die, but we have yet to reach a comprehensive understanding of whether and how protein stability is altered in surviving cells after IR exposure. 

## 5. Conclusions

Limited polysome profiles suggest a more profound extent of regulation at the level translation than at the level of transcription following exposure to ionizing radiation. However, we have yet to know how widespread this finding is and what the consequence to the proteome is. Synthesis counter-balanced by degradation could produce protein-to-protein variations in the effect of IR, with many of these changes resulting in functional consequences. Proteome-wide analysis of protein levels coupled with polysome profiles will supplement transcriptome datasets and will help us reach a more complete understanding of responses to IR.

## Supplementary Materials

Supplementary File 1

## Abbreviations

4E-BP1: 4E binding protein 1
ATM: ataxia telangiectasia mutated
CAF: cancer associated fibroblast
DDR: DNA damage responses
DSB: double strand break
eEF: eukaryotic elongation factor
EGFR: epidermal growth factor receptor
eIF: eukaryotic Initiation Factor
ERLIC: electrostatic repulsion-hydrophilic interaction chromatography, a method for enriching phosphor-peptides
ESI: Electron Spray Ionization, a technique to ionize molecules for mass spectrometry, especially useful with macromolecules
GEF: guanine nucleotide exchange factor
HuR: Hu antigen R, also known as ELAVL1
ICPL: isotope coded protein labeling, typically applied to tissue lysates
IR: ionizing radiation
IRES: internal ribosome entry site
ITAF: IRES transacting factors
JNK: Jun N-terminal kinase
LC: liquid chromatography for physical separation of molecules
LC-MS: a combination of LC with mass spectrometry
MALDI-TOF: matrix assisted laser desorption/ionization-time of flight, a mass spectrometry technique
MDM2: mouse double minute 2
PABP: poly A binding protein
PDGF: platelet-derived growth factor
PI3K: phosphatidylinositol 3-kinase
PIC: pre-initiation complex
SILAC: stable isotope labeling by/with amino acids in cell culture
TOR: target of rapamycin
UTR: untranslated region
VEGF: vascular endothelial growth factor
