# Supplementary figures and images for: The Role of Translational Regulation in Survival after Radiation Damage; an Opportunity for Proteomics Analysis
Source: Proteomes. 2014 Jun 11;2(2):272–90. doi: 10.3390/proteomes2020272 (PMC4530795; doi:10.3390/proteomes2020272)

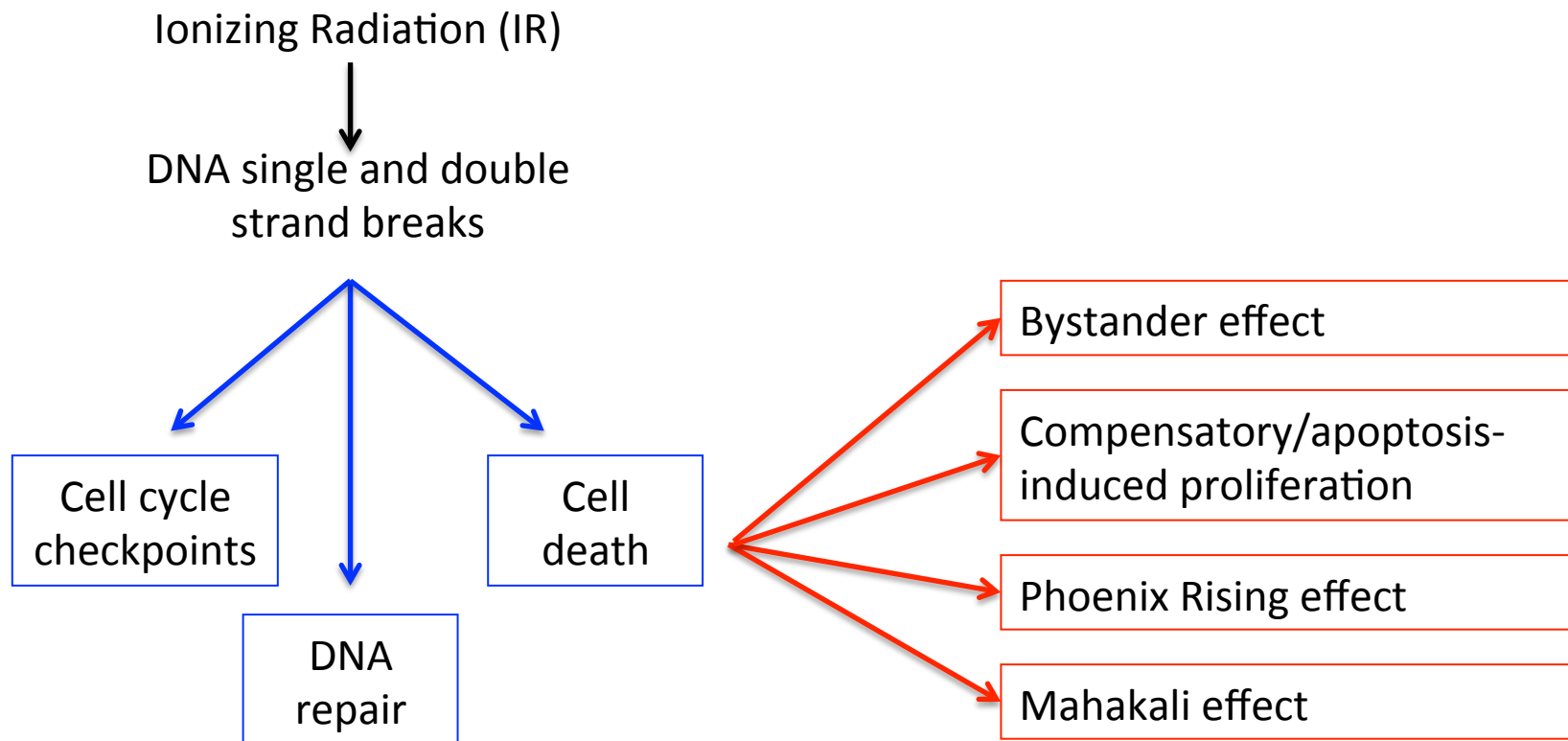

Stickel, Gomes and Su, Figure 1

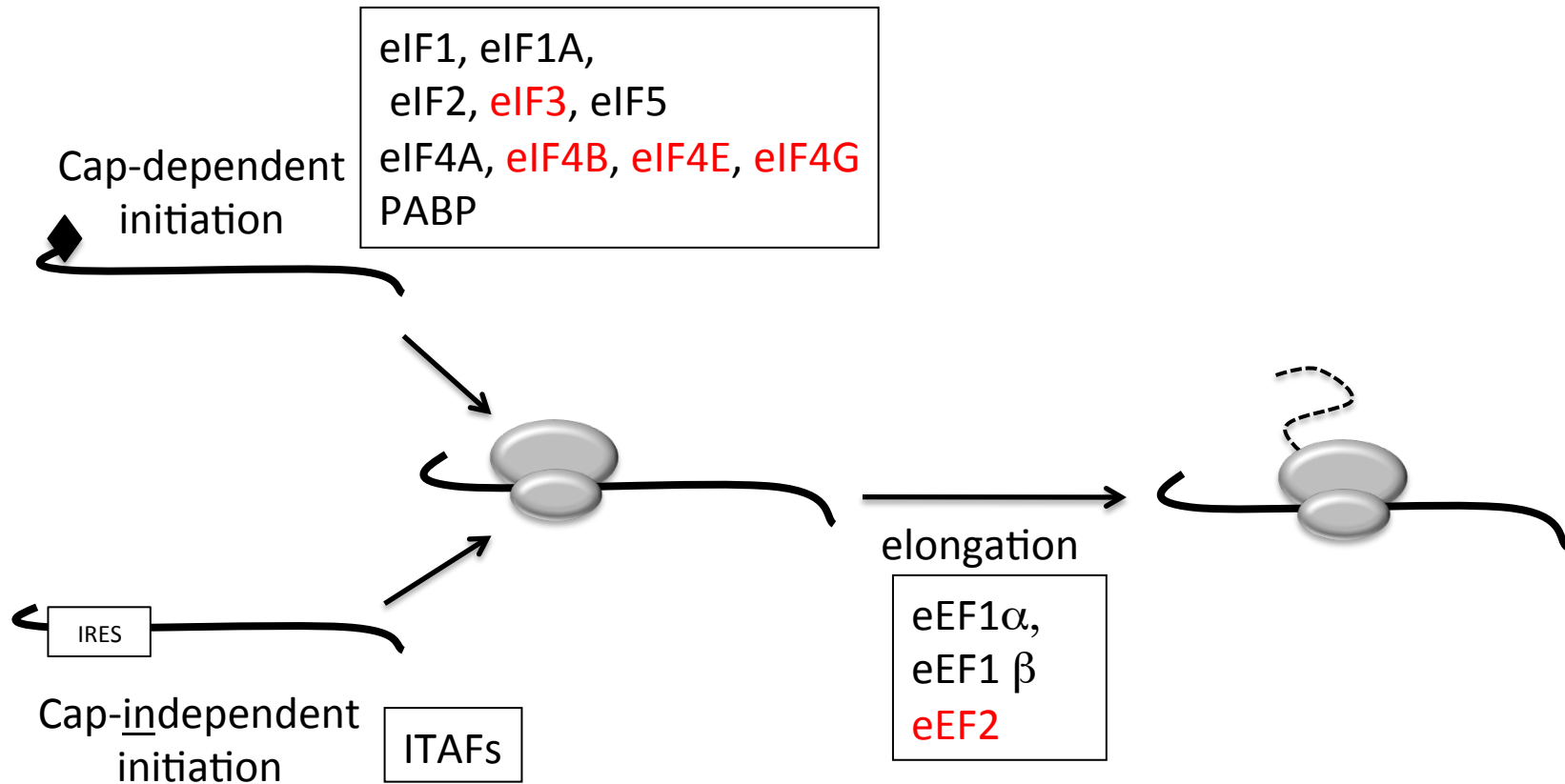

Stickel, Gomes and Su, Figure 2

Supplement: Supplementary File 1 [file proteomes-02-00272-s001.pdf]
